# Ethical considerations in an era of mass drug administration

**DOI:** 10.1186/1756-3305-6-234

**Published:** 2013-08-09

**Authors:** Barney Wharam, Luke Lazarou

**Affiliations:** 1 School of Biological Sciences, University of Bristol, Woodland Road, Bristol BS8 1UG, UK

**Keywords:** Drug resistance, Mass drug administration, Ethics, Neglected tropical diseases, The value of life

## Abstract

In a Plenary debate at the 51st Spring meeting of the British Society of Parasitology, Bristol, UK, April 8–11, 2013, the bioethicist James Wilson used the value of a life in the present and future to question the effectiveness of current health strategies.

## Main text

A debate during the 51st spring meeting of the British Society of Parasitology, Bristol, UK, April 8–11, 2013, considered the broader ethical and practical issues of current strategies used in controlling parasites worldwide.

For thousands of years humans have attempted to treat the infections that were ubiquitous in their communities. With the advent of improved sanitation and advances in medicine, parasitic infections rarely impact on human life in the developed world. This contrasts with the developing world where to be infected is still normal in many communities. In sub-Saharan Africa at least 500 million people are affected by Neglected Tropical Diseases (NTDs). The current control strategy in sub-Saharan Africa involves large multi-million dollar programmes of mass drug administration, in which whole populations are treated regardless of whom is infected. Such widespread, prolonged use of chemotherapy comes with its own problems. What is the best long-term answer for humanity to address such a huge burden of disease? Do we attempt to treat these parasites through the same means as we did in the developed world, or are there other, better ways?

### Ethical considerations in treating disease

Is a life in the future worth less than a life in the present? James Wilson (University College London) pointed out that many current health strategies implicitly assume that saving a life now is more important than saving a life in the future, for example until recently the World Health Organisation’s Global Burden of Disease project discounted the value of future lives. The logic of these health strategies normally involves the uncertainty of the future. Because we have no real way of predicting the state of future populations, unforeseen events such as war or emerging diseases may make investments in the future worthless.

Despite this, are current policy makers really making decisions that benefit the most people irrespective of when they live? If all human life is valued equally, healthcare resources should ideally be used in a way that maximises the health benefit for the largest number of people. Wilson highlighted the fact that if present and future lives were given equal weight, then perhaps the most ethical course of action would be to invest most of our resources now to eradicate diseases, where possible, and therefore to save countless future lives. Even if attempts at eradication of a parasitic infection might ultimately fail, then this is not a reason not to try, because the benefits of success are so large. Furthermore, even failed attempts at eradication can lead to large populations being relieved of their burden of disease if only temporarily.

### Drug resistance and the future

Programmes that aim to eradicate parasites through chemotherapy have their own risks in the form of drug resistance. The evolution of widespread drug resistance in parasites population both renders a drug impotent and reduces our ability to treat disease. This is especially problematic if there are only one or a few drugs effective against a parasite. The emerging problem of antibiotic resistance was used as a current, real world example of drug resistance by both Wilson and Andrew Read (Pennsylvania State University).

Read illustrated the problems of drug resistance through his recent visit to a Michigan hospital, where patients are dying from “superbugs” - bacteria that are resistant to many or all antibiotics that are available. Patients alive now are put into jeopardy because of actions from the past. Antibiotic resistance demonstrates how past health care policies did not sufficiently account for the evolution of parasites and therefore discounted the value of future lives inappropriately.

Read argued that there is a lack of research on how to use drugs whilst minimising the evolution of drug resistance. Drug resistance is also a key issue in veterinary science and the fields of human and veterinary medicine have become so distinct that veterinary studies are rarely used to inform human medicine. Movements such as The One Health Initiative (http://www.onehealthinitiative.com/index.php) were highlighted by Read as a possible way forward. The initiative aims to improve global healthcare through interdisciplinary collaborations.

### The era of mass drug administration

Alan Fenwick (Imperial College and Director of the Schistosomiasis Control Initiative) summarized the major global initiatives to control or eliminate NTDs, which has led to four major pharmaceutical companies (GlaxoSmithKline, Merck, Pfizer and Johnson & Johnson) donating substantial quantities of drugs for the control of 5 NTDs. Fenwick explained that the control and treatment of onchocerciasis has virtually eliminated blindness due to onchocerciasis from West Africa. However, for this success to be maintained, 100 million people must be treated annually.

If mass drug administration programmes succeed in breaking cycles of transmission they can be effective in controlling and perhaps even eradicating diseases. Even if eradication is not possible it may still be effective at reducing the burden of disease for future generations. However, Read argued that anthelminthic drugs will eventually fail due to resistance evolving, pointing to previous failures of antibiotics, antimalarial drugs and pesticides. The World Health Organization current policy is that drugs will eventually fail. Read urged us not to think of drugs as magic bullets but instead to realise that they are stop-gap measures.

Perhaps the question is not will resistance develop, but actually can we use strategies that make these drugs last until other factors eliminate disease? A comment from the audience raised the point that these diseases may never be properly controlled until we have effective sanitation and hygiene in the affected communities. Perhaps a more integrated approach will be successful in the long term whilst benefitting people now. As an example of this, Fenwick highlighted a recent Bill and Melinda Gates Foundation Conference, “Finding Synergies between Water, Sanitation, and Hygiene (WASH) and the Control of Neglected Tropical Diseases (NTDs): Practical Considerations to Collaboration Between the WASH and NTD Sectors.”

If each and every human life is of equal value we should all have access to treatment. If a life in the future and a life in the present are of equal value then we need to act in a way that treats people now as well as being responsible to people in the future. If withdrawing treatment from people alive now actually benefitted more lives in the future should we do it? The reality is that we are not able to predict the future. Can we justify stopping mass drug administration on the basis of unquantifiable risk? How can we change mass drug administration to minimise that risk?

## Authors’ contributions

BW and LL drafted the manuscript; both authors read and approved the final manuscript.

